# Integrative Analysis on the Urinary Proteome of Diabetic Kidney Disease, with an Emphasis on Extracellular Matrix Proteins

**DOI:** 10.3390/ijms27052283

**Published:** 2026-02-28

**Authors:** Sonnal Lohia, Jerome Zoidakis, Antonia Vlahou, Aggeliki Tserga

**Affiliations:** 1 Center of Systems Biology, Biomedical Research Foundation of the Academy of Athens, 11527 Athens, Greecevlahoua@bioacademy.gr (A.V.); 2 Department of Biology, National and Kapodistrian University of Athens, 30 Panepistimiou Street, 15772 Athens, Greece

**Keywords:** DKD, urine, proteomics, transcriptomics, matrisome, proteins, biomarkers, extra-cellular matrix, ECM proteins

## Abstract

One of the key pathological features of Diabetic Kidney Disease (DKD) progression is the accumulation of extracellular matrix (ECM) proteins in kidneys, leading to thickening of the glomerular and tubular basement membranes, subsequently resulting in mesangial expansion, sclerosis, and tubulointerstitial fibrosis. Given the high prevalence of DKD among both T2DM and T1DM patients, as well as the complexity of its underlying molecular mechanisms, this study provides a comparative analysis of published urinary proteomics datasets in DKD (*n* = 4). By integrating these data with published tissue proteomics (*n* = 2) and published transcriptomics datasets (*n* = 5), the study further aims to link urinary findings to tissue pathophysiology. Through integrative proteomic and transcriptomic analysis, DKD was associated with distinct alterations in the urinary proteome, particularly involving proteins related to ECM turnover. Using multiple validation datasets, several upregulated proteins with potential biological significance were identified, including annexins, collagens, cathepsins, and glycoproteins. Overall, our findings underscore the critical role of ECM remodeling in DKD progression and further validation could open new avenues for biomarker development and targeted therapy in early stages of DKD.

## 1. Introduction

Diabetes mellitus (DM) is a systemic metabolic disorder associated with significant morbidity and mortality. According to the 11th edition of the International Diabetes Federation (IDF) Atlas, in 2025, diabetes affects 11.1% of the adult population (20–79 years) corresponding to 588 million individuals, and by 2050, this number is expected to rise to approximately 852.5 million adults [1]. Diabetic kidney disease (DKD) includes individuals diagnosed with type 1 (T1DM) or type 2 diabetes (T2DM), who exhibit functional abnormalities in the kidneys as a result of the diabetes progression [2,3], which eventually further leads to higher rates of morbidity and mortality in DM patients. Not surprisingly, the global prevalence of DKD is also rising, with an estimated 10–40% of T2DM patients and 30% of T1DM patients developing DKD [4].

DKD is clinically diagnosed based on the presence of persistent proteinuria, reduced estimated glomerular filtration rate (eGFR) and an albumin-to-creatinine ratio > 30 mg/g [5,6]. Interestingly, unlike other chronic kidney diseases (CKD), DKD does not always show a direct correlation between declining eGFR and increasing albuminuria. In fact, some DKD patients may transition from microalbuminuria to normoalbuminuria over time [7,8]. This observation highlights the need for more sensitive biomarkers than microalbuminuria for the early detection and monitoring of DKD progression.

One of the key pathological features of DKD progression is the accumulation of extracellular matrix (ECM) proteins in the kidneys. This leads to thickening of the glomerular and tubular basement membranes, and subsequently resulting in mesangial expansion, sclerosis, and tubulointerstitial fibrosis [5,9,10]. Given their significant role in the disease, several studies have proposed that measuring ECM protein levels in urine could serve as a potential non-invasive biomarker for early detection of DKD [11].

Proteomic approaches play a pivotal role in biomarker discovery, permitting large-scale analysis of proteins in biological samples [12]. Since the cellular and tissue proteome constantly change under physiological conditions, comparing urinary proteomic profiles of DKD patients to non-diabetic and diabetic controls can help identify early diagnostic markers and reveal mechanisms of DKD progression [12,13,14]. A characteristic example is CKD273, the first proteomic-based classifier successfully used for the prognosis of CKD progression [15] utilized in the PRIORITY trial [5] for the early detection of DKD, also providing further insight into its pathophysiology [5].

Multiple peptidomic datasets have associated early ECM changes to DKD [16,17,18]. Given the high prevalence of DKD, and the complexity of its underlying molecular mechanisms, this study performs a comparative analysis of published urinary proteomics datasets in DKD and integrates them with renal tissue proteomics and transcriptomics data, in order to link urinary changes to kidney pathophysiology. We hypothesize that early DKD is characterized by dysregulation of ECM remodeling pathways, reflected by consistent alterations in urine matrisome-related proteins.

## 2. Results

### 2.1. Identification of DEPs

We conducted an integrative analysis to investigate protein changes in urine of individuals with DKD in an integrative manner, aiming to increase the power and coverage of the biological processes it reflects, particularly with respect to ECM alterations. The first cross-sectional study in the discovery cohort [19], to be referred to as Study 1 henceforth, was composed of urine samples, including DKD patients (*n* = 65), and CKD patients (*n* = 40, CKD without T2DM). A total of 2946 proteins at high confidence levels were identified using the LC-MS/MS and data-dependent acquisition analysis (DDA). On comparison of DKD vs. non-diabetic control group, 582 statistically significant proteins (*p* < 0.05) were identified, out of which 474 DEPs were upregulated and 93 DEPs were downregulated in DKD.

The second exploratory study in the discovery cohort [20], to be referred as Study 2 henceforth, was composed of urine samples from 36 patients with T2DM (which were classified into normal albuminuria, microalbuminuria, and macroalbuminuria groups) and 12 healthy subjects. As reported in the published study, proteomic analysis of the 48 urine samples was performed using a data-independent acquisition approach, yielding the identification of a total of 1004 proteins at high confidence levels across all disease groups. For our analysis, we combined the microalbuminuria and macroalbuminuria groups into a single group called DKD (*n* = 24), which was compared with the healthy control group (*n* = 12). On comparison of the DKD vs. healthy controls group, 790 statistically significant proteins were identified (*p* < 0.05), out of which 652 DEPs were upregulated and 131 DEPs were downregulated in the former versus the latter.

### 2.2. Pathway and PPI Network Analysis of DEPs

Targeting to identify underlying molecular mechanisms associated with DKD at increased coverage, a union of DEPs identified from Study 1 and Study 2 was subsequently used. A total of 181 upregulated (Table S2) and 44 downregulated (Table S2) DEPs were identified when comparing DKD versus non-diabetic controls. To explore the biological relevance of these DEPs, pathway enrichment analysis was conducted using the Metascape platform (version v3.5.20250101; https://metascape.org/, accessed on 26 May 2025), based on the GO Biological Processes and Reactome Gene Sets databases. The results (Table S2) highlight that these proteins are predominantly involved in pathways related to ECM formation including fibrin clot formation, wound healing, and plasminogen activation.

**Figure 1:**
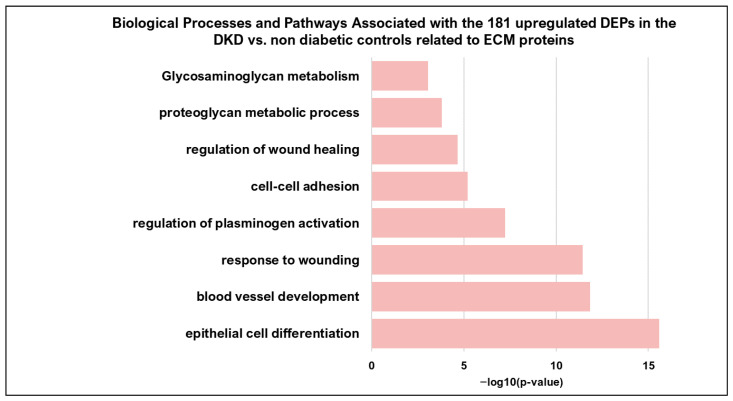
Selected enriched pathways and processes associated with the 181 upregulated DEPs, detected in the comparison of DKD vs. non-diabetic controls comparison (Metascape) and are related to ECM formation/organization, cell adhesion, plasminogen activation, regulation of wound healing and tissue development. DEP, differentially expressed protein.

Specifically, of the 181 upregulated proteins, 60 DEPs were associated with Matrisome, out of which 29 are included in the Matrisome DataBase (http://matrixdb.univ-lyon1.fr/, accessed on 10 June 2025) [21] and 31 are related to ECM, according to the literature. These proteins are associated with the regulation of cell adhesion, plasminogen activation, ECM regulation, regulation of wound healing and tissue development, as illustrated in Figure 1 and further detailed in Table S3.

To identify the most significant clusters of DEPs, a PPI network analysis of the 181 upregulated proteins was generated and analyzed, as depicted in Figure S1. Among the nine most significant modules identified using Cytoscape’s MCODE plug-in (Cytoscape App version 3.9 with MCODE version 2.0.3), the module associated with ECM degradation is detected, as shown in Table S2. Among these modules, a total of 10 Matrisome hub proteins were identified, including Annexin A6 [P08133; ANXA6] (MCODE1), Annexin A11 [P50995; ANXA11] (MCODE3), Annexin A2 [P07355; ANXA2] (MCODE3), Protein S100-A8 [P05109; S100A8] (MCODE3), Protein S100-A9 [P06702; S100A9] (MCODE3), Collagen alpha-1(XV) chain [P39059; COL15A1] (MCODE4), Collagen alpha-1(VI) chain [P12109; COL6A1] (MCODE4), Collagen alpha-3(VI) chain [P12111; COL6A3] (MCODE4), Sphingosine-1-phosphate phosphatase 1 [Q9BX95; SPP1] (MCODE4) and Thrombospondin-1 [P07996; THBS1] (MCODE4) highlighted in Figure S1.

Besides ECM, additional pathways and modules are predicted based on the observed urine changes in DKD patients related to immune response and cellular homeostasis, included in Table S2.

### 2.3. Validation of Predictions

#### 2.3.1. Validation Through Proteomics Datasets

We further compared our predictions of Matrisome and Matrisome-related proteins with the protein expression data included in the validation urinary and tissue datasets, representing T1DM or T2DM DKD.

In Study 3, Zhao et al. [22] examined the transcriptomic and proteomic profiles of kidney biopsies from T2DM DKD patients comparing them to those of normal controls. We further compared the relative changes in protein and gene expression reported in this study with the deregulated proteins predicted in our analysis (Table 1). Out of the 60 Matrisome DEPs identified as significantly altered in DKD in our datasets, 22 were differentially expressed and at consistent expression trends in Zhao et al. [22]. These included core matrisome and matrisome-associated proteins according to the Matrisome Database [21] such as Galectin-3 [P17931; LGALS3], COL15A1, Tetranectin [P05452; CLEC3B], COL6A1, Thrombospondin-1 [P07996; THBS1], COL6A3, Dipeptidyl peptidase 1 [P53634; CTSC], Plasma serine protease inhibitor [P05154; SERPINA5], Annexin A3 [P12429; ANXA3], Lysosomal protective protein [P10619; CTSA], ANXA6, Multimerin-2 [Q9H8L6; MMRN2], Fibroleukin [Q14314; FGL2], ANXA11, Mannan-binding lectin serine protease 2 [O00187; MASP2], Annexin A1 [P04083; ANXA1] and Matrisome-related proteins based on literature search, such as Brain acid soluble protein 1 [P80723; BASP1], Extracellular superoxide dismutase [Cu-Zn] [P08294; SOD3], Leukocyte surface antigen CD47 [Q08722; CD47], Galectin-3-binding protein [Q08380; LGALS3BP], Arylsulfatase A [P15289; ARSA], and Basigin [P35613; BSG]. Consistencies were also observed at the mRNA level for 20 urinary DEPs including Collagen alpha-1(XV) chain [P39059; COL15A1], LGALS3, Urokinase-type plasminogen activator [P00749; PLAU], Protein S100-A6 [P06703; S100A6], Angiopoietin-related protein 2 [Q9UKU9; ANGPTL2], COL6A3, CTSC, COL6A1, ANXA1, ANXA3, ANXA6, Cathepsin D [P07339; CTSD] and Matrisome-related proteins (according to the literature) including the Immunoglobulin superfamily containing leucine-rich repeat protein [O14498; ISLR], Lysosomal acid phosphatase [P11117; ACP2], BASP1, SOD3, Pancreatic adenocarcinoma upregulated factor [Q96DA0; ZG16B], Cell division control protein 42 homolog [P60953; CDC42], G-protein coupled receptor family C group 5 member B [Q9NZH0; GPRC5B], and Peroxiredoxin-1 [Q06830; PRDX1].

In addition, we further evaluated the protein expression data from Hirohama et al., [23] referred to as Study 4 in Table 1, which involved proteomics analysis of the kidney tissue of 23 T2DM DKD patients and 10 healthy controls. Analyzing their proteomics dataset generated using the SomaScan platform, we identified five proteins (ANXA1, ANXA2, SOD3, Kininogen-1 [P01042; KNG1] and LGALS3BP) as upregulated, in agreement with our findings from the discovery cohort.

Of particular note is Study 5, [24], involving a urinary proteomics study of young T1DM patients compared to healthy controls. These youths were in the earliest and uncomplicated stage of DKD. Upon reanalysis of the raw data, we identified five Matrisome proteins (CTSD, CTSA, CTSC, ARSA, and Alpha-N-acetylglucosaminidase [P54802; NAGLU]) as upregulated in patients compared to controls, further supporting the relevance of our discovery cohort findings.

In a complementary analysis, Musante et al., [25] (Study 6) investigated the expression levels of 34 proteases and 32 protease inhibitors in urinary extracellular vesicles (UEVs) from urine samples of T1DM DKD patients divided in normo-, micro- and macroalbuminuric groups, compared to healthy controls. We compared their reported protein expression levels of proteases and protease inhibitors with those observed in our dataset (Table 1). A subset of proteases (CTSA, CTSC, CTSD, Dipeptidyl peptidase 4 [P27487; DPP4] and PLAU) showed progressive increases across the DKD groups compared to healthy controls in line with our findings. Additionally, the protein inhibitor SERPINA5 showed an increase in microalbuminuric group compared to controls.

#### 2.3.2. Tissue Expression and Cross-Omics Correlations in Nephroseq Database

According to the literature, and the Human Protein Atlas database (https://www.proteinatlas.org/, accessed on 28 May 2025), among the 60 Matrisome-related DEPs, 56 DEPs are already related to diabetes and/or DKD (highlighted in Table 1 and further in detail in Table S3), and 53 DEPs are linked to kidney expression and/or kidney function (Table 1 and in detail in Table S3). To further place our predictions in the context of published transcriptomics datasets in DKD and compile relevant evidence, kidney transcriptomics data from DKD patients vs. healthy controls were retrieved from the Nephroseq database (https://www.nephroseq.org, accessed on 30 May 2025) and seven datasets in total were included [26,27,28,29]. In addition, a single-cell kidney transcriptomic dataset of early human DKD [30] was also investigated. We detected a total of 42 Matrisome-related transcripts overlapping with the 60 Matrisome DEPs (Table 1 and Table S3). The direction of differential expression identified in the analysis of discovery cohort, for 31 (out of 60) Matrisome DEPs agreed with their respective mRNA expression trend in DKD, as observed in Nephroseq. However, for 11 (out of 60) Matrisome DEPs, the direction of differential expression identified in the discovery cohort analysis contrasted with that observed at the mRNA levels in DKD by Nephroseq (Table 1 and Table S3). However, in the single-cell kidney transcriptomic dataset from early human DKD, three (Heat shock 70 kDa protein 1B [P0DMV9; HSPA1B], Extracellular sulfatase Sulf-2 [Q8IWU5; SULF2] and LGALS3) out of the 18 Matrisome DEPs that were not detected in the Nephroseq databases, showed consistent mRNA expression trends with that obtained from our analysis of the discovery cohort.

**Table 1:** Summary of the expression of the 29 Matrisome DEPs (core matrisome and matrisome-associated proteins according to the Matrisome Database [21]) as identified in the discovery cohort, validated by transcriptomic kidney studies in the Nephroseq database and the proteomics validation cohorts. FC: Fold Change. Only statistically significant (*p*-values < 0.05) results are presented in the Table; *p*-values are provided in Table S3. Empty cells correspond to proteins or mRNAs that were either not detected or not differentially expressed in the corresponding study.

Gene Symbol/Protein Name	Discovery Cohort	Validation Cohort			
Study	(Study 1/Study 2)		Study 3	Study 4	Study 6
Dataset Type	Urine Proteomics [19,20]	Tissue Transcriptomics Nephroseq Datasets (DKD vs. Controls) [26,27,28,29]	Tissue Proteomics and Transcriptomics [22]	Tissue Proteomics [23]	Urine Proteases and Protease Inhibitors in UEVs [25]
ANGPTL2/ Angiopoietin-related protein 2	FC = 5.4/FC = 10.52		mRNA (FC = 2.04)		
ANXA1/ Annexin A1	FC = 2.78/FC = 32	DB5: FC = 1.55 DB4: FC = 2.46 DB6: FC = 2.48 DB2: FC = 5.74 DB3: FC = 2.67	protein (FC = 1.17)/mRNA (FC = 1.8)	FC = 2.14	
ANXA11/ Annexin A11	FC = 5.76/FC = 5.59	DB1: FC = −1.54	protein (FC = 1.32)		
ANXA2/ Annexin A2	FC = 3.67/FC = 9.93	DB4: FC = 1.87 DB6: FC = 1.95 DB2: FC = 3.6 DB3: FC = 2.3		FC = 1.3	
ANXA3/ Annexin A3	FC = 4.87/FC = 14.5	DB5: FC = 1.71 DB4: FC = 1.83 DB6: FC = 1.76 DB2: FC = 4.13 DB3: FC = 2.28	protein (FC = 2.9)/mRNA (FC = 1.72)		
ANXA4/ Annexin A4	FC = 3.33/FC = 3	DB4: FC = 1.53 DB2: FC = 2.17 DB1: FC = 1.51			
ANXA6/ Annexin A6	FC = 2.38/FC = 4	DB5: FC = 1.55	protein (FC = 1.7)/mRNA (FC = 1.46)		
CLEC3B/ Tetranectin	only in DKD/FC = 4.6		protein (FC = 6.57)		
COL15A1/ Collagen alpha-1(XV)	FC = 1.54/FC = 9.59	DB5: FC = 4 DB6: FC = 1.7 DB2: FC = 3.98 DB1: FC = 2.14 DB3: FC = 3.7	protein (FC = 6.65)/mRNA (FC = 3.57)		
COL6A1/ Collagen alpha-1(VI)	FC = 2.63/FC = 32.68	DB3: FC = −1.77	protein (FC = 5.4)/mRNA (FC = 1.89)		
COL6A3/ Collagen alpha-3(VI)	FC = 17.03/FC = 3.34	DB5: FC = 5.56 DB4: FC = 1.86 DB6: FC = 2.56 DB1: FC = 4.68 DB2: FC = 5.19 DB3: FC = 4.28	protein (FC = 5.07)/mRNA (FC = 1.94)		
CTSA/ Lysosomal protective protein/Cathepsin A	FC = 3.44/FC = 10.84		protein (FC = 2.34)		FC = 4.34
CTSC/ Dipeptidyl peptidase 1/Cathepsin C	FC = 2.18/FC = 12.45	DB5: FC = 2.05	protein (FC = 1.4)/mRNA (FC = 1.89)		FC = 2.5
CTSD/ Cathepsin D	FC = 1.34/FC = 5.69	DB2: FC = 1.51 DB3: FC = 1.55	mRNA (FC = 1.18)		FC = 6.88
ELANE/ Neutrophil elastase	FC = 1.45/FC = 39.86				
FGL2/ Fibroleukin	FC = 5.17/FC = 4.41	DB5: FC = 2.04 DB4: FC = 2.96 DB6: FC = 2.17 DB2: FC = 2.83 DB3: FC = 2.43	protein (FC = 1.4)		
KNG1/ Kininogen-1	FC = 1.72/FC = 6.83	DB5: FC = −2.26 DB6: FC = −1.618 DB2: FC = −5.08 DB3: FC = −2.02		FC = 1.76	
LGALS3/ Galectin-3	FC = 3.53/FC = 2.66		protein (FC = 19.4)/mRNA (FC = 2.46)		
MASP2/ Mannan-binding lectin serine protease 2	FC = 2.49/FC = 8.35	DB3: FC = −3.94	protein (FC = 1.18)		
MMRN2/ Multimerin-2	FC = 8.13/FC = 9.5	DB3: FC = 1.6	protein (FC = 1.65)		
MUC1/ Mucin-1	FC = 1.73/FC = 14.16	DB4: FC = 1.59 DB2: FC = 1.95 DB1: FC = 1.7 DB3: FC = 2.01			
PLAU/ Urokinase-type plasminogen activator	FC = 2.21/FC = 18	DB5: FC = 1.83	mRNA (FC = 2.31)		FC = 1.11
S100A6/ Protein S100-A6	FC = 8.51/FC = 7.22	DB4: FC = 1.68 DB3: FC = 2.31	mRNA (FC = 2.28)		
S100A8/ Protein S100-A8	FC = 10.2/FC = 58.07	DB3: FC = 5.85 DB5: FC = 1.88 DB6: FC = 2.23 DB2: FC = 2.28			
S100A9/ Protein S100-A9	FC = 10.1/FC = 45.37	DB6: FC = 1.57 DB3: FC = 11.2			
SERPINA5/ Plasma serine protease inhibitor	FC = 2.26/FC = 7.95		protein (FC = 3.14)		FC = 1.32
SPP1/ Osteopontin	FC = 16.4/FC = 178.3	DB2: FC = 1.97 DB1: FC = 2.01			
SULF2/ Extracellular sulfatase Sulf-2	FC = only in DKD/FC = 4.45				
THBS1/ Thrombospondin-1	FC = 16.31/FC = 1.9	DB4: FC = 1.91 DB2: FC = 3.43 DB3: FC = 1.63	protein (FC = 5.36)		

DB1: Woroniecka Diabetes Glom [29]; DB2: Woroniecka Diabetes TubInt [29]; DB3: ERCB Nephrotic Syndrome TubIn; DB4: Schmid Diabetes TubInt [27]; DB5: Ju CKD Glom [26]; DB6: Ju CKD TubInt [28].

## 3. Discussion

DKD is a microvascular complication affecting approximately 20% to 50% of patients with DM and is a leading cause of end-stage kidney disease (ESKD), contributing substantially to increased morbidity and mortality [31]. However, reduced eGFR and albuminuria, although commonly used, are not specific biomarkers for DKD [6], and early kidney injury may develop during an asymptomatic phase despite normoalbuminuria and normal GFR [32].

Major histopathological features of diabetic nephropathy include mesangial ECM expansion, thickening of the glomerular and tubular basement membranes, tubular atrophy, and interstitial fibrosis, all of which directly contribute to disease pathogenesis [33]. Consistent with these changes, DKD is characterized by an increased deposition of glomerular matrix proteins, including collagens (I, III, IV, and V), and fibronectin, as well as ECM glycoproteins such as laminin and proteoglycans [5,18]. Hyperglycemia is a major driver of ECM accumulation, mediated through upregulation of TGF-β signaling. Dysregulated ECM turnover also promotes fibrosis through an imbalance between matrix metalloproteinases (MMPs) and their inhibitors (TIMPs), leading to impaired ECM degradation and progressive collagen accumulation [34]. Increased ECM turnover in DKD and dysregulation of ECM-related proteins (for example: collagens), promotes abnormal remodeling and excessive ECM deposition, with increased release of these proteins into biological fluids such as urine during disease progression [35,36].

This study aimed to investigate urinary proteomic alterations related to ECM and associated with DKD and its progression. A union of DEPs identified from two available datasets [19,20] formed the discovery cohort and was used for further analysis. Specifically, we focused on 181 statistically significant upregulated proteins in DKD versus non-diabetic controls, obtained by combining the statistically significant proteins between the two studies. Pathway enrichment and PPI network analyses of these proteins highlighted ECM-related processes, including ECM formation, tissue development and wound healing (Figure S1). Among the enriched pathways highly relevant to the pathophysiology of DKD were inflammation and disruption of cellular homeostasis, both closely linked to ECM production. Chronic inflammation contributes to aberrant wound-healing responses, characterized by fibroblast activation and excessive ECM production, which promote glomerulosclerosis and tubulointerstitial fibrosis [37,38]. Similarly, disruption of cellular homeostasis, including mitochondrial dysfunction and oxidative stress, promotes ECM accumulation, which further increases kidney injury [39,40]. Changes in the relative abundance of ECM and ECM-related proteins in the urine of DKD patients, compared to non-diabetic controls, may reflect alterations in ECM deposition or turnover in the diabetic kidney. Among the 181 DEPs, a total of 60 Matrisome DEPs were identified. Out of these, 10 were predicted as hub proteins in the PPI network analysis, including annexins (ANXA11, ANXA2, ANXA6), collagens (COL15A1, COL6A1, COL6A3), calprotectin subunits (S100A8, S100A9), and glycoproteins (Sphingosine-1-phosphate phosphatase 1 [Q9BX95; SPP1], and Thrombospondin-1 [P07996; THBS1]), as shown in Figure S1.

The annexin family of calcium-dependent phospholipid-binding proteins has a significant, although sometimes dual, role in the pathogenesis and progression of DKD. Key members include Annexin A1 (ANXA1) and Annexin A2 (ANXA2), which have been implicated in inflammation, fibrosis, and renal structural changes. ANXA1 plays important roles in the innate immune response as an effector of glucocorticoid-mediated responses and a regulator of the inflammatory process [41]. In a recent study, mRNA and protein levels of ANXA1 in the glomeruli from renal biopsy samples were detected as increased in patients with DKD compared with healthy controls [22]. Interestingly, Ka et al. demonstrated that ANXA1 protein was expressed in 52.94% of DKD patients with normoalbuminuria [42]. Thus, it is suggested that urinary ANXA1 may serve as an early non-invasive diagnostic marker for glomerular injury. ANXA2 is a plasminogen receptor and tissue plasminogen activator, promoting plasmin generation and regulation of the homeostasis of blood coagulation and matrix balance [43]. Increased ANXA2 expression is associated with abnormal cell proliferation, enhanced inflammation and the promotion of auto-antibody renal injury [44]. ANXA2 in vitro silencing blocked collagen VI secretion and alleviated glomerular injury, which suggests that ANXA2 participates in the progression of DKD [45].

Annexin A6 (ANXA6) is also a calcium-dependent phospholipid-binding protein. Although its exact role in diabetic kidney disease (DKD) is still under investigation, ANXA6′s upregulation has been found to promote renal damage, specifically in the context of calcium-related oxidative stress injury, suggesting a possible contribution to the structural changes observed in diabetic kidneys [46]. ANXA6 interacts with heterotetrameric calprotectin (S100A8/A9) and associates with tubulin filaments in activated monocytes [47]. S100A8 and S100A9 are calcium- and zinc-binding proteins with a prominent role in the regulation of inflammatory processes and immune response [48]. In a recent study, Du et al. [49] reported a significant upregulation of S100A8 and S100A9 in tubular epithelial cells of diabetic kidneys which promoted renal interstitial fibrosis through the TLR4/NF-κB signal pathway. Interestingly, treatment with the small molecule inhibitor AB38b, which targets the abnormal expression of these proteins, may offer a promising therapeutic strategy for mitigating renal interstitial fibrosis associated with DKD [49].

Among the hub proteins, three collagens were highlighted. Collagen alpha-1(XV) chain (COL15A1) has been previously detected as upregulated at the gene level in the glomeruli of DKD patients compared to healthy controls, suggesting a potential involvement in the altered composition of the endothelial surface layer (ESL). However, its low co-localization with glomerular endothelial markers indicates it may play a more limited or indirect role in the glomerular ESL compared to other proteoglycans [50]. COL15A1 was also detected as upregulated in both DKD and hepatocellular carcinoma (HCC) patients and was identified as a key hub gene linking the two conditions. It may serve as a diagnostic biomarker for DKD-associated HCC and is potentially involved in immune cell infiltration, suggesting a role in inflammation-driven tumor progression [51]. Collagen VI (ColVI) is minimally expressed in healthy adult kidneys but plays a structural role at the glomerular basement membrane interface. In diabetes, its deposition increases significantly, replacing COLIV in nodular lesions. In a recent study, COL6A1 and COL6A3 (as part of COL VI) are implicated in all-cause mortality in T2DM patients with microalbuminuria [47].

Among the hub genes there are two glycoproteins, SPP1 and THBS1. Osteopontin (SPP1/OPN) is a highly phosphorylated glycoprotein that regulates various biological processes, including immune activation, and chronic inflammation [52]. It is also known as early T-lymphocyte activation 1 protein, and interacts with integrins and CD44 to influence cell signaling and ECM interactions [53]. Diabetes increases SPP1 gene expression in mesangial kidney cells [54], and overexpression of SPP1 potentially leads to glomerular damage in a mouse model of DKD [55]. In T2DM patients, plasma SPP1 level was significantly correlated with the degree of DKD [56]. In urine, an increased SPP1 excretion level was detected in DKD patients compared to diabetic [57] or non-diabetic controls [58]. In the latter study, urinary SPP1 levels were elevated in diabetic albuminuric and nephrotic syndrome patients compared to controls and normoalbuminuric individuals, suggesting its potential as an early diagnostic marker. This increase could reflect glomerular dysfunction caused by diabetic renal injury and may serve as an indicator of kidney function. THBS1 is a multifunctional protein mediating cell-to-cell and cell-to-matrix interactions [59], involved in inflammation, angiogenesis, wound healing and metabolic homeostasis [60]. Previous studies indicated that glomerular protein and mRNA THBS1 expression increases with progression of DKD comparing DKD to non-diabetic control patients [61].

In addition to the identified hub proteins, six proteins (ANXA1, CTSA, CTSC, CTSD, PLAU and LGALS3BP) that were upregulated at the protein or mRNA level in at least three validation datasets are also of high biological relevance, as observed in the Nephroseq database [26,27,28,29]. Lysosomal protective protein (CTSA), Dipeptidyl peptidase 1 (CTSC) and Cathepsin D (CTSD) are proteases, members of the family of lysosomal cathepsins. Although associated with tissue fibrosis, these proteins have not been extensively studied in the context of DKD, with the exception of CTSD. CTSA is a protective protein, essential for both the activity and stability of beta-galactosidase and neuraminidase [62]. Ahn et al. [63] proposed CTSA, as one of five urinary proteins that significantly improved the prediction of DKD prognosis in comparison to urinary albumin concentration or eGFR. CTSC acts as both an exopeptidase and endopeptidase with a key role in immune and inflammatory response [64]. In the study by Audzeyenka et al., [65] inhibition of CTSC reverses podocyte damage induced by high glucose, whereas the protein was detected at increased levels in the urine of obese Zucker rats with kidney damage. CTSD plays a role in intracellular protein degradation and is implicated in mediating inflammation and atherosclerosis [66]. Given its role in collagen degradation and the metabolism of angiotensinogen to angiotensin, increased expression and activity of CTSD is expected in DKD [67]. Its synthesis is stimulated by insulin and has been proposed as a novel biomarker for T2DM risk and insulin resistance (IR) [68]. Interestingly, urinary CTSD protein levels are detected as elevated in DKD patients compared to healthy or diabetic controls [18,25,67,69]. Limonte et al., [67] in a case–control study using four T1DM cohorts, suggested that urine CTSD is associated with rapid eGFR decline in T1DM and reflects kidney tubulointerstitial injury. Interestingly, in the re-analysis of a urinary proteomics study of young T1DM patients compared to healthy controls (Study 5) [24], CTSA, CTSC and CTSD were identified as upregulated in patients compared to controls. As such, these three cathepsins could be of great significance for the early detection of DKD.

Besides cathepsins, Urokinase-type plasminogen activator (PLAU/uPA) is another protease, specifically, serine protease involved in tissue remodeling and cell migration [70]. It cleaves the zymogen plasminogen to form the active enzyme plasmin. Johnson et al. recently detected that PLAU levels are significantly elevated in the serum of DKD patients compared to healthy controls. Their findings also demonstrated a moderate diagnostic performance (AUC ≈ 0.75 in discriminating DKD) [70]. DKD has been linked to elevated urinary levels of plasmin, prostasin, and urokinase, leading to proteolytic activation of epithelial sodium channel (ENaC), which may impair renal sodium excretion and contribute to hypertension [71]. Proteases potentially have a key role in the pathogenesis of DKD, and further research is needed to better understand their impact on the disease due to their complexity.

Galectin-3-binding protein (LGALS3BP) is a secreted multifunctional glycoprotein whose expression is stimulated under inflammatory conditions. It interacts with members of the ECM, such as integrins and fibronectins [72,73]. As such, it has been studied for its regulatory role in innate immunity and multiorgan fibrosis [72,73,74]. To our knowledge, no studies have specifically investigated the role of LGALS3BP in DKD. However, its expression is elevated in three human kidney proteomics and transcriptomics studies (Table 1). These findings suggest a potential immunogenic and fibrotic role of LGALS3BP in DKD, although its function remains unclear.

While this analysis provides valuable insights; it also has limitations. The low number of eligible studies reflects both the need for further investigation of early DKD and the importance of adhering to FAIR principles to enable data reuse. In particular, only two urinary DKD discovery and validation datasets were available with appropriate metadata for re-analysis. Although 15 additional relevant research articles were identified, their data were either not deposited in public repositories or were not reusable due to missing metadata and/or supporting files; moreover, attempts to obtain these data by contacting the authors were unsuccessful. Hence, the overall difficulty in accessing datasets of sufficient metadata for interoperability is an issue of concern [75]. Finally, observed discrepancies between urinary proteomics and renal transcriptomics profiles may reflect biological differences between protein and mRNA expression, as mRNA levels do not always correlate with protein abundance, likely attributing to post-transcriptional regulation, differential translation, and protein stability.

## 4. Materials and Methods

### 4.1. Study Search

A thorough review of the literature was performed in the following web sources and databases: PubMed, Google Scholar, and the ProteomeXchange repositories (http://www.proteomexchange.org, accessed on 28 May 2025), including PRIDE (https://www.ebi.ac.uk/pride/, accessed on 28 May 2025), MassIVE (https://massive.ucsd.edu, accessed on 28 May 2025), the Japan ProteOme STandard Repository (https://repository.jpostdb.org/, accessed on 28 May 2025), and the integrated Proteome Resources (https://www.iprox.cn/, accessed on 28 May 2025). Specifically, the keywords used in this search were “proteom*” AND “urine” AND “DKD” (it should be noted that additional keywords were tested in each case, such as instead of “DKD”—“diabetic kidney disease’’ OR “diabetic nephropathy’’, etc. were used, with overlapping results). All published (including peer-reviewed and preprint) research articles, written in English language from 1 January 2015 to 31 August 2024 were collected. Two authors (S.L. and A.T.) manually screened the abstracts and reviewed the full texts of the retrieved articles independently, and no discrepancies were found.

### 4.2. Selection Criteria for Discovery Cohort

**Figure 2:**
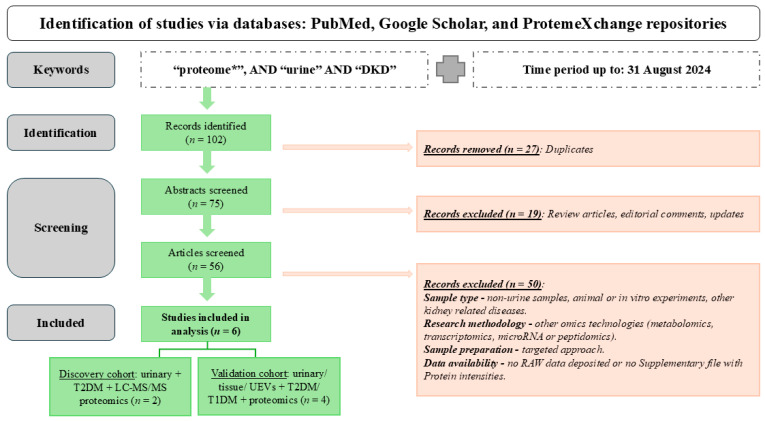
Workflow followed for the retrieval of the presented omics studies associated with urine proteomics and DKD. Additional keywords used for the search include “diabetic kidney disease’’ OR “diabetic nephropathy’’, etc., but yielded in most extent overlapping results. *—keywords were used with “*” to perform a broader search, irrespective of their use in multiple forms, for example plurals.

The search, as illustrated in Figure 2, yielded a total of 102 publications after excluding duplicate articles (since the search was performed on different web platforms, the same article was found multiple times). Predominantly, studies analyzing the urinary proteome of DKD diagnosed patients, were included in this analysis. Diagnosis was based either on the presence of albuminuria and/or reduced eGFR without signs of other primary causes of renal damage, or on pathological confirmation. Review articles, editorial comments, and updates on published research articles were removed, following the initial screening of abstracts. After a thorough review of the remaining articles, additional studies were excluded, namely studies that did not analyze urine samples, and/or focused on animal or in vitro experiments.

According to the IDF Atlas [1], T2DM is the most common type of diabetes mellitus, which was also reflected by the number of articles retrieved on T2DM (*n* = 97 articles) and T1DM (*n* = 5 articles) during our literature search. Next, the research methodologies were critically reviewed. Studies employing other omics technologies (such as metabolomics, transcriptomics, microRNA or peptidomics) as well as those not employing Liquid Chromatography coupled with Mass Spectrometry (LC-MS/MS) based proteomic approaches (e.g., enzyme linked immunosorbent assay (ELISA), Two-Dimensional Gel Electrophoresis coupled with Electrospray Ionization Time-of-Flight Mass Spectrometry (2D GE-ESI-TOF-MS, MALDI-TOF), Olink, SOMAScan, targeted MS based approaches like Multiple Reaction Monitoring (MRM) or Selected Reaction Monitoring (SRM), Protein Chip array) were further excluded from this analysis for the discovery cohort but were used for validation.

As above-mentioned, the retrieved studies were separated to a discovery and validation cohort mainly driven by technology (e.g., LC-MS/MS studies on urine proteome formed the discovery cohort whereas data collected on tissue proteomics formed the validation cohort). In addition, to increase consistency, only studies addressing T2DM were included in the discovery set, with analyses focusing on T1DM urinary proteomics being included in the validation cohort (described also below). Subsequently, a major issue encountered during the literature search was the lack of publicly available raw proteomics data. Additionally, many studies did not provide supplementary data, listing all identified proteins along with their respective intensities in each sample, making re-analysis impossible.

### 4.3. Validation Through Kidney Tissue Proteomics Datasets

To increase understanding and further validate our findings, proteomics datasets from DKD patients with T1DM and T2DM were also retrieved. This first validation cohort, (to be referred to as Study 3 henceforth) [22] consisted of glomerular tissue samples isolated from 50 biopsy-proven DKD patients and 25 age- and gender-matched non-diabetic control subjects (tissues were obtained from tumor-free tissues which were greater than 5 cm away from the surgical margin or histologically unaffected regions of tumor nephrectomies from non-diabetic patients). Proteomic analysis for this cohort was performed using LC-MS/MS and raw files were deposited in the PRIDE database with the identifier PXD040617. A second validation cohort, (to be referred to as Study 4 henceforth) was identified, in which the kidney proteome of T2DM patients was analyzed using an alternative proteomics platform, SOMAscan [23]. In this second validation cohort, an unbiased aptamer-based proteomic analysis was conducted on human kidney samples from 23 individuals with DKD, diagnosed based on eGFR levels < 60 mL/min/1.73 m^2^ in T2DM patients, and 10 healthy controls. Briefly, the assay utilized slow off-rate modified DNA aptamers (SOMAmers), which bind to 1305 specific protein targets. Protein levels were measured in relative fluorescence units, and the intensity of protein signals was transformed into base-2 logarithmic (log_2_) values, as provided in the Supplementary Files of the study.

### 4.4. Validation Through Urinary T1DM Dataset

To further validate the specificity of the findings related to T2DM, we identified an LC-MS/MS based proteomic study analyzing the urinary proteome of T1DM patients [24]. This third validation cohort, (to be referred to as Study 5 henceforth) consisted of 30 urine samples collected from otherwise healthy youths (≤19 years old), with (*n* = 15) and without (*n* = 15) T1DM. All participants were free of significant comorbidity including hypertension and proteinuria; and did not use corticosteroid, anti-hypertensive, or anti-inflammatory medications. Youths with T1DM were in the earliest and uncomplicated stage of the natural history of DKD, as reported in the published study. Proteomic analysis was performed using LC-MS/MS, and the raw files were deposited in the PRIDE database under the identifier PXD017213. All the proteomics datasets used for discovery and validation are listed in Table 2.

### 4.5. Protein Identification

Raw files (.raw) obtained from LC-MS/MS (Study 1 [19], Study 3 [22] and Study 5 [24]) were analyzed by Proteome Discoverer 1.4 using the SEQUEST search engine against the entire reviewed non-redundant human (accessed in April 2024) database downloaded as a FASTA file from UniProt. Cysteine carbamidomethylation was selected as fixed modification and methionine/proline oxidation and alkylation were selected as dynamic modifications. Trypsin was selected as the digestive enzyme. During the search, a precursor mass tolerance of 10 ppm, two missed cleavage sites, peptide length of 6–144 amino acids, and 0.05 Da fragment mass tolerance were permitted, with the false discovery rate (FDR) set stringently at 0.01, yielding a high confidence output. Of note, for the Study 2 [20], only author-provided processed outputs were available and were used for downstream comparative analyses.

### 4.6. Statistical Analysis

Statistical analysis was performed using the R programming software (R version 4.2.0 with 216 IDE: R Studio Version 1.2.5, Boston, MA, USA). For each of the study, the precursor ion area values as defined by Proteome Discoverer 1.4, were merged using an in-house R script and were subjected to a global (per sample) normalization as per the formula: X′ = (X/sum(Xi))  × 10^6^; where X is the raw protein area; sum(Xi) is the sum of all raw protein areas of a given sample; and X′ is the normalized protein area. The non-parametric Mann–Whitney U test was utilized for defining statistical significance. The *p*-values below 0.05 (*p* < 0.05) with Fold-change (FC) ratio > 1.3 for upregulation and FC ratio < 0.67 for downregulation were applied to define differentially expressed proteins (DEPs).

### 4.7. Bioinformatics Analysis

#### 4.7.1. Pathway Enrichment Analysis

Pathway enrichment analysis was performed using the Metascape platform (version v3.5.20250101; https://metascape.org/, accessed on 26 May 2025) [76]. The Gene Ontology (GO)—Biological Processes (BP) database was used for enrichment analysis, excluding pathways containing fewer than three genes. All other settings were kept at default, and significantly enriched pathways were defined by Benjamini–Hochberg-adjusted *p* < 0.05 [77]. Results were simplified based on biological relevance and selected terms are presented.

#### 4.7.2. Protein–Protein Interaction (PPI) Network Creation

To establish a PPI network of DEPs, Metascape platform (version v3.5.20250101; https://metascape.org/, accessed on 26 May 2025) [76] was used to retrieve interacting proteins. All protein–protein interactions among input proteins were extracted from PPI data source and formed a PPI network. Gene Ontology (GO) enrichment analysis was applied to the network to extract “biological meanings”. Molecular complex detection (MCODE) algorithm (using the Cytoscape App version 3.9 with MCODE version 2.0.3) was then applied to this network to identify neighborhoods where proteins are densely connected. In the PPI network, nodes indicate proteins and the edges represent functional or physical associations between proteins with a medium confidence interval (0.40). The default parameters for DEP clustering and scoring were set as follows: degree cutoff = 2, node score cutoff = 0.2, max depth = 100, and k-score = 2.

#### 4.7.3. Cross-Omics Confirmation

The list of DEPs in the form of EntrezGene IDs, was uploaded in Nephroseq v5 (https://www.nephroseq.org, accessed on 30 May 2025), a data resource consisting of comprehensive tools for analyzing kidney transcriptomics data. DKD dataset selection was conducted by the application of the following filters: Primary Filters > Group > Diabetic Nephropathy. Seven available DKD human datasets involving comparison of DKD vs. Healthy Living Donor groups were retrieved (Table S1). Only significantly deregulated genes (*p* < 0.05) were extracted, and their differential expression was compared with the DEPs.

## 5. Conclusions

Nowadays, there is growing effort to identify new biomarkers for early detection of DKD. The PRIORITY-trial [5] demonstrated that the urinary proteomic-based risk marker CKD273 is associated with early progression of DKD, providing added value to urinary albumin excretion and eGFR. Moving one step further, the present study focuses on total proteome instead of differentially expressed urinary peptides in DKD patients compared to non-diabetic controls. This first integrative analysis in urinary proteomics of DKD suggests that DKD is associated with distinct alterations in the urinary proteome, particularly involving proteins related to ECM turnover. Through integrative proteomic and transcriptomic analysis, using multiple validation datasets, several upregulated proteins were identified, including annexins, collagens, cathepsins, and glycoproteins, with potential diagnostic and biological significance. The identification of understudied proteins like ANXA11, CTSA, CTSC and LGALS3BP further expands the landscape of potential contributors to DKD pathogenesis. Overall, while, the findings of this integrative analysis underscore the critical role of ECM remodeling in DKD progression, further research and experimental validation are needed. Such efforts could open new avenues for biomarker development and therapeutic targeting in early stages of DKD.

## Figures and Tables

**Table 2:** Summary of proteomic studies included in the discovery and validation cohorts.

Study	Cohort	Sample Type	Instrument	Disease Model	Disease Group (Sample Size)	Control Group (Sample Size)
Study 1 [19]	Discovery	Urine	LC-MS/MS	DKD with T2DM versus CKD	DKD (*n* = 63)	CKD Non-diabetic (*n* = 40)
Study 2 [20]	Discovery	Urine	LC-MS/MS	DKD with T2DM versus Healthy controls	DKD (*n* = 24)	Healthy controls (*n* = 12)
Study 3 [22]	Validation	Glomerular tissue	LC-MS/MS	DKD with T2DM versus Non-Diabetic controls	DKD (*n* = 50)	Control (*n* = 25)
Study 4 [23]	Validation	Kidney Tissue	SOMAscan	DKD with T2DM versus Healthy controls	DKD (*n* = 23)	Healthy controls (*n* = 10)
Study 5 [24]	Validation	Urine	LC-MS/MS	T1DM youths versus Healthy youths	T1DM (*n* = 15)	Healthy controls (*n* = 15)
Study 6 [25]	Validation	Urine (UEVs)	Proteome profiler human protease and protease inhibitor array kits	T1DM versus Healthy controls	T1DM (*n* = 37)	Healthy controls (*n* = 12)

## Data Availability

The original contributions presented in this study are included in the article/Supplementary Materials. Further inquiries can be directed to the corresponding authors.

## Supplementary Materials

The following supporting information can be downloaded at: https://www.mdpi.com/article/10.3390/ijms27052283/s1.

## Abbreviations

The following abbreviations are used in this manuscript:

DM	Diabetes mellitus
IDF	The International Diabetes Federation
DKD	Diabetic kidney disease
T1DM	Type 1 Diabetes mellitus
T2DM	Type 2 Diabetes mellitus
eGFR	Estimated Glomerular Filtration Rate
CKD	Chronic Kidney Diseases
ECM	Extracellular matrix
LC-MS/MS	Liquid Chromatography coupled with Mass Spectrometry
ELISA	Enzyme linked immunosorbent assay
2D GE-ESI-TOF-MS	Two-Dimensional Gel Electrophoresis coupled with Electrospray Ionization Time-Of-Flight Mass Spectrometry
MRM	Multiple Reaction Monitoring
SRM	Selected Reaction Monitoring
FDR	False Discovery Rate
FC	Fold-change Ratio
DEPs	Differentially Expressed Proteins
PPI	Protein–protein interaction
GO	Gene Ontology
MCODE	Molecular Complex Detection Algorithm
