# Supplementary material for: Integrative Analysis on the Urinary Proteome of Diabetic Kidney Disease, with an Emphasis on Extracellular Matrix Proteins
Source: Int J Mol Sci. 2026 Feb 28;27(5):2283. doi: 10.3390/ijms27052283 (PMC12985236; doi:10.3390/ijms27052283)
Supplement: Supplementary file 1 [file ijms-27-02283-s001.zip › ijms-4100816-Supplementary-Figure S1.pdf]

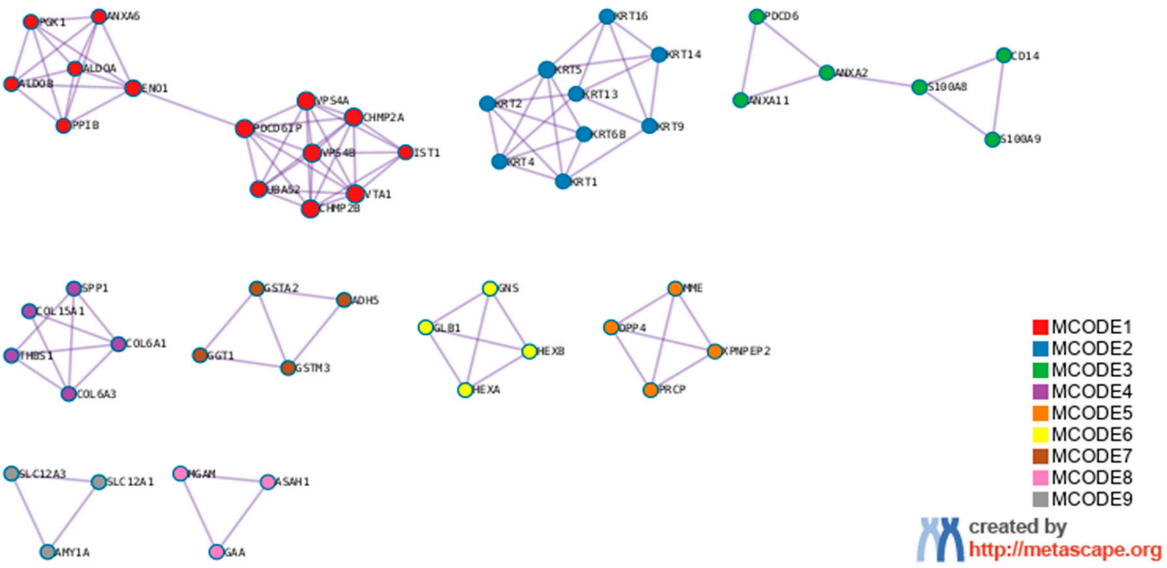

Figure S1. PPI MCODE Components. MCODE algorithm was applied to the PPI network to identify neighborhoods where proteins are densely connected. Its MCODE cluster is colored separately. MCODE1: ESCRT III complex disassembly, MCODE2: Intermediate filament organization, MCODE3: Regulation of TLR by endogenous ligand, MCODE4: Degradation of the extracellular matrix, MCODE5: Protein maturation, MCODE6: Glycosaminoglycan catabolic process, and MCODE7: Glutathione metabolic process.
